# Successful baroreflex activation therapy in a case of therapy-resistant renovascular hypertension

**DOI:** 10.1007/s00392-022-02137-5

**Published:** 2022-12-22

**Authors:** Eva Maria Nuhn, Manuel Wallbach, Florian Elger, Michael Johann Koziolek

**Affiliations:** 1 Department of Nephrology and Rheumatology, University Medical Centre, Robert-Koch-Str. 40, 37075 Göttingen, Germany; 2 German Center for Cardiovascular Research (DZHK), Partner Site Göttingen, Robert-Koch-Str. 30, 37075 Göttingen, Germany; 3 Department of Cardiac, Thoracic and Vascular Surgery, University Medical Centre, Robert-Koch-Str. 40, 37075 Göttingen, Germany

Sirs:

The ASTRAL, CORAL, and STAR intervention trials failed to show a benefit of stent angioplasty in patients with atherosclerotic renal artery stenosis (aNAS) regarding overall survival, blood pressure and/or kidney function compared with optimal antihypertensive drug therapy and cardiovascular risk factor control [1]. Revascularization is associated with a reduced risk of cardiovascular events and reduced progression of end-stage renal disease in high-risk patients with pulmonary oedema, severe hypertension, or rapidly deteriorating renal function [2]. However, best practice in high-risk patients with aNAS and contraindication for stent angioplasty is currently unclear.

The following case report describes the successful use of baroreflex activation therapy (BAT) in a high-risk patient with resistant hypertension and aNAS in whom neither interventional nor surgical revascularization could be performed.

 A 73-year-old female patient presented in our hypertension center with systolic blood pressure (BP) up to 200 mmHg and sensation of cephalic pressure. There were no angina pectoris symptoms, no dyspnoea, and no syncopal episodes. The patient’s past medical history included peripheral arterial occlusive disease, pulmonary artery embolism, and history of smoking (25 pack years).

**Fig. 1:**
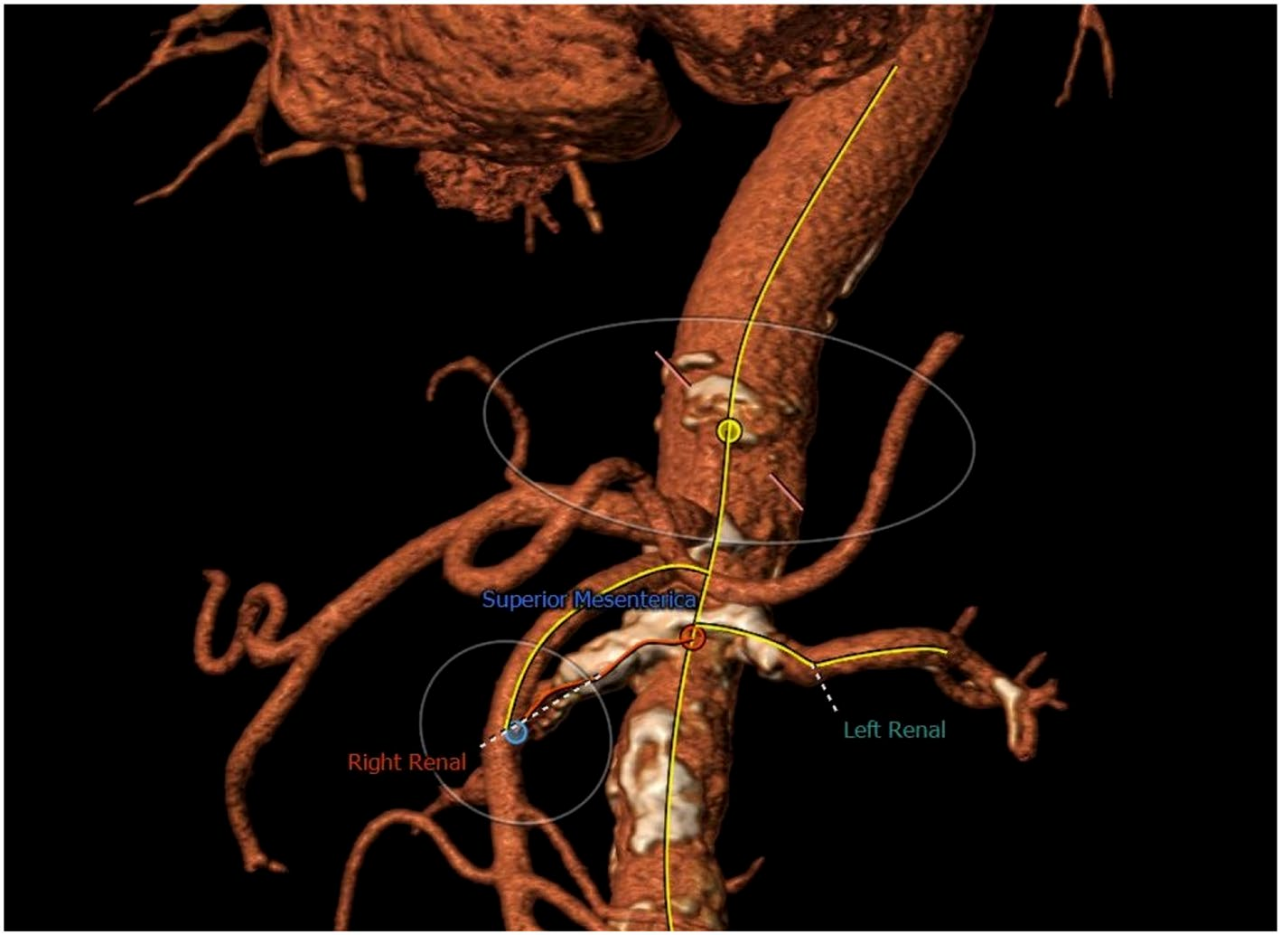
3D CT scan reconstruction of patients´ individual anatomy showing coral reef aorta

 The patient exhibited a marked difference in office blood pressure (BP) between the right (124/74 mmHg) and the left arm (160/74 mmHg). The arm with the higher BP was used for all subsequent readings. CT angiography showed severe atherosclerosis of the aorta (coral reef aorta, Fig. 1), stenoses of the coronary arteries, high-grade stenosis of the distal right subclavian artery as well as higher-grade calcified stenoses of the proximal left subclavian artery, the juxtarenal aorta, and the renal arteries on both sides. Extracranial duplex sonography revealed a 30–35% stenosis of the proximal internal carotid artery according to NASCET criteria (= 60% ECST) and a consecutive stenosis of the left external carotid artery. Renal perfusion and function scintigraphy disclosed reduced and inhomogeneous perfusion of the right kidney. On the left side, perfusion was normal with no evidence of obstruction. In addition, other causes of secondary hypertension have already been excluded in outpatient care in accordance with the guidelines [4, 6], including determination of plasma metanephrines and normetanephrines, aldosterone/renin quotient, TSH, and sleep apnea screening.

Long-term BP measurement, performed according to guideline [4], showed an average of 165/69 mmHg in the daytime and 148/62 mmHg in the nighttime, equaling nocturnal BP dipping of 10.4%. For pharmacological treatment of the elevated BP, medication with an ACE inhibitor was started with gradual uptitration in the follow-up with intake of whole medication under supervision to ensure full therapy adherence during inpatient stay. This was followed by a consecutive increase in serum creatinine from 0.96 mg/dl (eGFR 57.8 ml/min/1.73 m^2^) to 1.76 mg/dl (eGFR 28.5 ml/min). Urinalysis did not reveal any conclusive findings. Antihypertensive therapy with ACE inhibitors, beta-blockers, diuretics, dihydralazine, calcium antagonists, and alpha2-receptor agonists did not result in adequate BP control (162/68 mmHg). Vascular interdisciplinary board recommended a renal artery revascularization by percutaneous transluminal renal angioplasty (PTRA) of the right renal artery, which was performed with a failed attempt due to a coral reef stenosis of the juxtarenal aorta and a complex stenosis of the target vessel. Surgical intervention would most likely have involved open replacement of the significantly wall-altered juxtarenal aorta and therefore appeared disproportionate and highly risky. Despite treatment with six antihypertensive drugs, the patient suffered from recurrent hypertensive urgencies with systolic BP above 180 mmHg. We therefore initiated BAT as a rescue therapy. Subsequent BP after activation of BAT revealed a significant reduction with average values of 133/61 mmHg. The renal parameters remained at a stable level with a creatinine value of 1.44 mg/dl (eGFR 36 ml/min) after six months of BAT. The time line of relevant findings is summarized in Table 1.

**Table 1:** Time course of patients´ BP and renal relevant parameters

	before BAT/at BAT initiation	6 months after BAT
Office BP (mmHg)	197/84	153/73
ABPM (mmHg)	162/86	133/61
Dipping (%)	10.39	4.74
numbers of antihypertensives (n)	6	7
Antihypertensive Therapeutic Index (ATI)	55.83	43.25
Antihypertensives and dosages	ACEi (Ramipril) 10 mg/d CCR (Lercanidipine) 10 mg/d Diuretics (Hygroton) 25 mg/d BB (Bisoprolol) 5 mg/d DV (Dihydralazine) 75 mg/d α2RA (Moxonidine) 0.8 mg/d	ACEi (Ramipril) 5 mg/d CCR (Amlodipine) 10 mg/d Diuretics (Torasemid) 5 mg/d BB (Bisoprolol) 5 mg/d DV (Minoxidil) 10 mg/d α2RA (Moxonidine) 0.6 mg/d AA (Spironolactone) 25 mg/d
Heart rate (BPM)	75/min	71/min
serum creatinine (mg/dl)	1.76	1.44
eGFR (ml/min)	28.5	36
albuminuria (mg/g creatinine)	29	11
pulse width (ms) amplitude (mV) frequency (sec^−1^)	125 6.2 40	125 4.8 45

*AA* aldosterone-antagonist, *ACEi *ACE inhibitor, *BB* betablocker, BPM = Beats per minute, *CCR* calcium channel antagonist, *α2RA* α2-receptoragonist, *DV* direct vasodilatator, ATI = [( dose of antihypertensive drug 1 / maximum dose of antihypertensive drug 1 + dose of antihypertensive drug 2 / maximum dose of antihypertensive drug 2 + dose of antihypertensive drug n /maximum dose of antihypertensive drug n) × 10]

 Currently, device-based hypertension therapy is not recommended as a routine treatment option according to ESC/ESH guidelines. [3] The use of BAT can be considered for the treatment of patients with resistant hypertension (level of evidence IIb, grade of recommendation C). [4] In addition to lowering arterial BP, BAT has potentially a protective effect on kidneys and the vascular system. [5] In the specific case of secondary arterial hypertension such as aNAS, the use of BAT is currently not recommended due to a lack of data, but also not explicitly contraindicated. [6] The case report presented here shows for the first time the successful use of BAT in a high-risk patient with aNAS after an unsuccessful attempt of revascularization. Treatment resulted in a relevant reduction in BP and stabilization of renal function. In unilateral renal artery stenosis, the renin-aldosterone system (RAAS) is activated in the healthy contralateral kidney. Volume-induced hypertension can largely be compensated by the healthy, non-stenosed kidney through pressure natriuresis with increased water excretion. [7] In addition, however, there is an increase in sympathetic nervous activity. [8] BAT modulates the autonomic nervous system by stimulating the baroreceptors near the carotid bifurcation with a consecutive decrease in sympathetic and activation of parasympathetic tone. [9] Our case report shows that in addition to its known beneficial effect in renoparenchymatous hypertension [10] BAT might also achieve a significant reduction in BP in renovascular hypertension. However, until more data is available, patients should only be selected for BAT in experienced centers and following current recommendations. [3]
